# Gigantol Targets Cancer Stem Cells and Destabilizes Tumors via the Suppression of the PI3K/AKT and JAK/STAT Pathways in Ectopic Lung Cancer Xenografts

**DOI:** 10.3390/cancers11122032

**Published:** 2019-12-17

**Authors:** Nattanan Losuwannarak, Arnatchai Maiuthed, Nakarin Kitkumthorn, Asada Leelahavanichkul, Sittiruk Roytrakul, Pithi Chanvorachote

**Affiliations:** 1 Cell-Based Drug and Health Product Development Research Unit, Faculty of Pharmaceutical Sciences, Chulalongkorn University, Bangkok 10330, Thailand; los.nattanan@hotmail.com (N.L.); m.arnatchai@gmail.com (A.M.); 2 Department of Pharmacology and Physiology, Faculty of Pharmaceutical Sciences, Chulalongkorn University, Bangkok 10330, Thailand; 3 Department of Oral Biology, Faculty of Dentistry, Mahidol University, Bangkok 10400, Thailand; nakarinkit@gmail.com; 4 Department of Microbiology, Faculty of Medicine, Chulalongkorn University, Bangkok 10330, Thailand; a_leelahavanit@yahoo.com; 5 Proteomics Research Laboratory, 113 Thailand Science Park, Phahonyothin Road, Khlong 1, Khlong Luang, Pathum Thani 12120, Thailand; sittiruk@biotec.or.th

**Keywords:** gigantol, AKT, JAK/STAT, cancer stem cell, tumor maintenance, tumor density, lung cancer, proteomics

## Abstract

Lung cancer has long been recognized as an important world heath concern due to its high incidence and death rate. The failure of treatment strategies, as well as the regrowth of the disease driven by cancer stem cells (CSCs) residing in the tumor, lead to the urgent need for a novel CSC-targeting therapy. Here, we utilized proteome alteration analysis and ectopic tumor xenografts to gain insight on how gigantol, a bibenzyl compound from orchid species, could attenuate CSCs and reduce tumor integrity. The proteomics revealed that gigantol affected several functional proteins influencing the properties of CSCs, especially cell proliferation and survival. Importantly, the PI3K/AKT/mTOR and JAK/STAT related pathways were found to be suppressed by gigantol, while the JNK signal was enhanced. The in vivo nude mice model confirmed that pretreatment of the cells with gigantol prior to a tumor becoming established could decrease the cell division and tumor maintenance. The results indicated that gigantol decreased the relative tumor weight with dramatically reduced tumor cell proliferation, as indicated by Ki-67 labeling. Although gigantol only slightly altered the epithelial-to-mesenchymal and angiogenesis statuses, the gigantol-treated group showed a dramatic loss of tumor integrity as compared with the well-grown tumor mass of the untreated control. This study reveals the effects of gigantol on tumor initiation, growth, and maintain in the scope that the cells at the first step of tumor initiation have lesser CSC property than the control untreated cells. This study reveals novel insights into the anti-tumor mechanisms of gigantol focused on CSC targeting and destabilizing tumor integrity via suppression of the PI3K/AKT/mTOR and JAK/STAT pathways. This data supports the potential of gigantol to be further developed as a drug for lung cancer.

## 1. Introduction

A new paradigm shift in the field of cancer cell biology is being driven by the concept of a key cancer cell population controlling the whole tumor, termed “cancer stem cells (CSCs)” [1]. CSCs from various types of cancers share a number of conservation properties, such as self-renewal ability, the generation of multiple types of differentiated cancer cells to drive tumor growth and heterogeneity, and resistance to chemotherapy via an upsurge of the DNA repair system and drug efflux transporter [2]. Therapeutic strategies targeting CSCs, including CSC direct eradication, CSC differentiation into tumor bulk cells, deletion of the supportive signals from a CSC niche, and suppression of CSC pathways, could lead to effective cancer therapy [3].

In lung adenocarcinoma, CSCs from patients were found to be less than 1.5% of the whole tumor cell population [4], but this small subpopulation was still substantial for tumorigenesis and tumor relapse [5]. The key driving pathways of CSCs, such as the PI3K/AKT/mTOR and JAK/STAT3 signals, were found to be significantly increased in cancers with high CSC properties, and hence investigations of many small molecules targeting such pathways are ongoing in clinical trials [6,7]. Protein kinase B (PKB) or AKT, which is, in fact, frequently upregulated in lung cancer plays a key role in cell survival and proliferation [8]. The activation of AKT was shown to be related with cisplatin resistance in lung cancer cells [9]. The roles of AKT on the properties of CSCs and their survival have been demonstrated in several key studies [10,11]. Likewise, signal transducer and activator of transcription 3 (STAT3) activation has been associated with poor prognosis as well as augmented CSCs [12]. A higher level of phosphorylated STAT3 (active STAT3) contributed to epithelial-to-mesenchymal transition (EMT) as well as increased CSC-like phenotypes of non-small cell lung cancer cells (NSCLCs), while the inhibition of STAT3 caused the opposite effects [13]. Instead of bulk non-CSC tumor clearance, the targeting of AKT and STAT3 is believed to be a promising anti-cancer strategy that could lead to the tumor collapse and prevention of the relapse of the disease [14,15].

**Figure 1:**
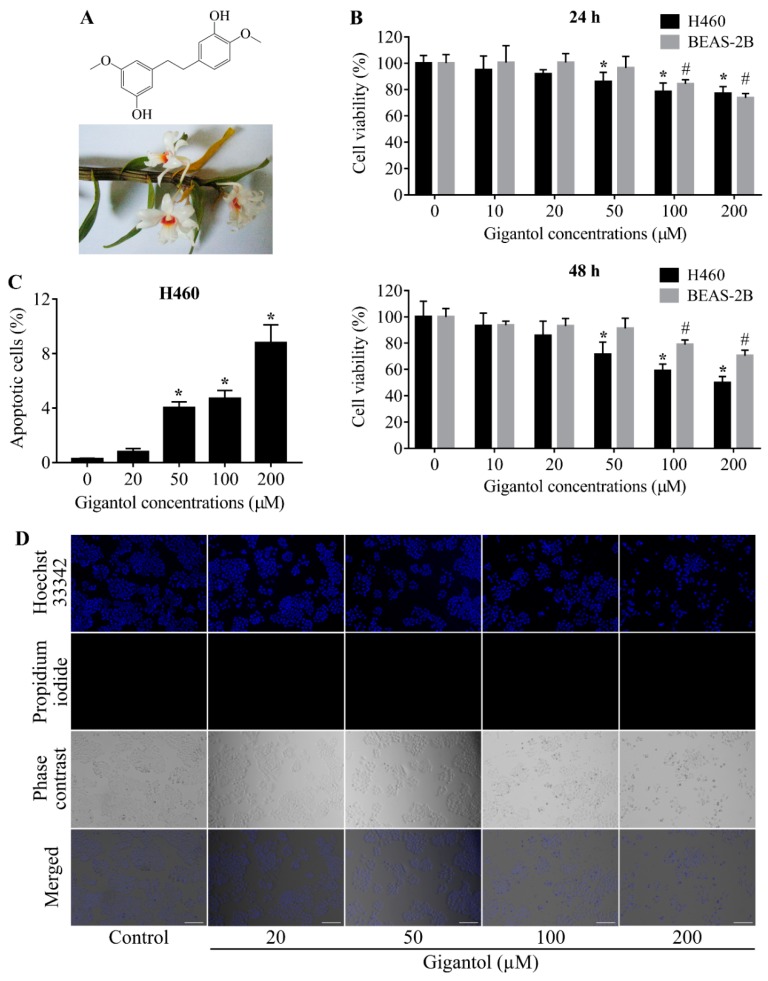
(**A**) Gigantol structure and the plant specimen, Dendrobium draconis Rchb.f. (**B**) Graphs showing the percentages of H460 and BEAS-2B cells viability. Both cell lines were treated with 0 to 200 µM of gigantol or vehicle for 24 to 48 h and then analyzed by MTT assay for cell viability. The percentages of viable cells were compared to their untreated controls. (**C**) Graph showing the percentages of apoptotic cell death after 24 h of gigantol exposure. Necrotic cells could not be detected. (**D**) Photographs of Hoechst 33342, Propidium iodide (PI), and phase contrast fields showing cancer cells morphologies after 24 h of gigantol treatment, the scale bars represent 100 µm and the magnification is 100×. Each experiment was performed in biological triplicates, * indicates *p* < 0.05 as compared with untreated group of H460, # indicates *p* < 0.05 as compared with untreated group of BEAS-2B (one-way ANOVA, Dunnett’s test).

Recently, natural compounds from plants have garnered increasing attention either as potential drugs or lead compounds in drug discovery research [16,17]. The key benefits of natural compounds are the abundance of plants, compound diversity, and cost effectiveness. In focusing on CSCs and tumor growth inhibition, previous studies have reported the promising activities of the bibenzyl derivative chrysotoxine in the suppression of AKT and Src [18]. In vivo studies further revealed that the bibenzyl derivative moscatilin reduced tumor volumes of lung and esophageal cancer xenografts [19,20]. Gigantol, a bibenzyl compound, is one of the polyphenolic components frequently found in traditional Chinese medicine, and has been shown to have several pharmacological effects, e.g., anti-inflammatory, amelioration of diabetic nephropathy and cataract, and anti-cancer [21,22,23]. The structure of gigantol consists of a bibenzyl core (Figure 1A). In vitro studies reported that gigantol triggered the apoptotic cell death of lung cancer cell lines but was not toxic to keratinocytes [24].

Our previous studies revealed several effects of noncytotoxic concentrations of gigantol on NSCLCs [25,26,27,28]. Pretreatment of 5 to 20 µM of gigantol showed a reduction of the tumor-forming capacity of NSCLCs, represented by significantly suppressing the anchorage-independent growth. In addition, with a single pretreatment of gigantol, the ability of cancer cells to form spheroids, a critical hallmark of CSCs, was abundantly suppressed [25]. These data indicated that the cancer cells had lost their self-renewal capability, which was confirmed by Western blot results showing the downregulation of octamer-binding transcription factor 4 (Oct 4) and Nanog, essential transcription factors for self-renewal and CSC-like phenotype maintenance [25]. Altogether, gigantol has the potential to attenuate tumorigenesis. However, certain information regarding the tumor growth attenuation mechanism and key evidence in animal models are still required. In this study, a subcutaneous xenograft model, as well as proteomic analysis of tumor growth regulatory pathways, were performed to help illustrate a clearer picture of how gigantol could suppress lung cancer.

## 2. Results

### 2.1. Determination of Noncytotoxic Concentrations of Gigantol

Treatment of human NSCLCs H460 with 10 to 20 µM of gigantol for 24 and 48 h had a nonsignificant effect on survival of the cells, while a significant reduction of cell survival could be first detected in response to gigantol at a concentration of 50 µM (Figure 1B). Moreover, cell viability evaluation revealed that gigantol exhibited less toxicity to human lung epithelial cells BEAS-2B as compared with lung cancer cells. Confirmation of cell death, either via apoptosis or necrosis, was detected under a fluorescent microscope after staining with Hoechst 33342 and propidium iodide (PI), as described in the Materials and Methods section. The nuclear staining results revealed that condensed and fragmented nuclei of apoptosis cells could be observed only in the cells treated with gigantol at 200 µM. It is worth indicating that treatment with gigantol at all concentrations (0 to 200 µM) caused no necrosis (Figure 1C,D). Noncytotoxic concentrations of gigantol (0 to 20 µM) were used in subsequent experiments.

### 2.2. Functional Classification and Enrichment Analysis of the Down- and Upregulated Proteins in Gigantol-Treated Cells

**Figure 2:**
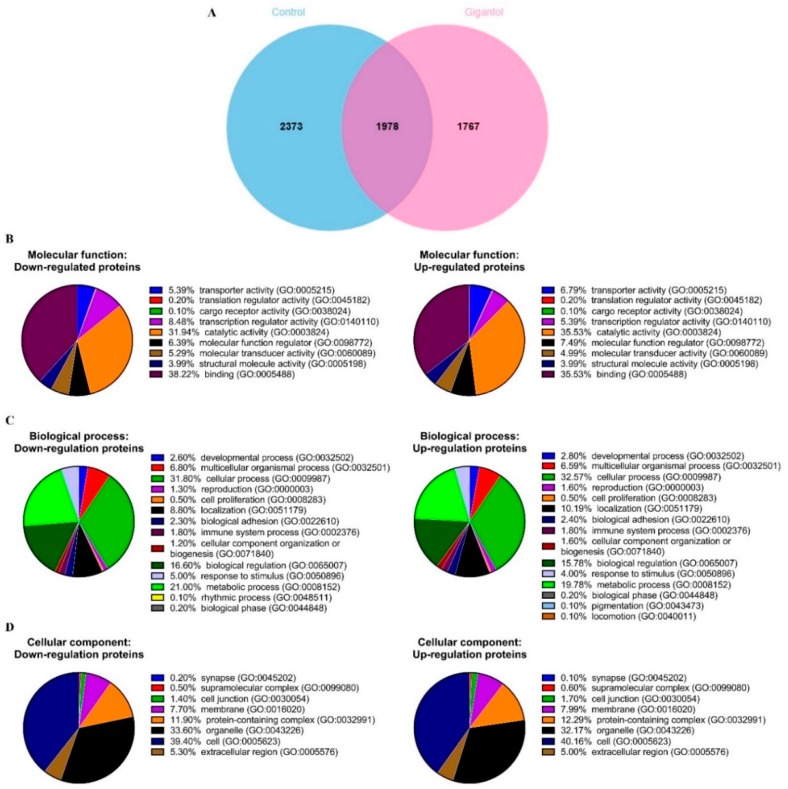
H460 cells were treated with 20 µM of gigantol or its vehicle (0.004% DMSO) for 24 h before the whole-cell lysates were collected. The experiment was performed in biological triplicates. (**A**) Venn diagram showing the difference in proteins expressions between the control and gigantol-treated H460 cells. Three functional classifications of the 2373 down- and 1767 upregulated proteins affected by gigantol treatment using Panther software: (**B**) molecular function, (**C**) biological process, and (**D**) cellular component.

In total, 4351 proteins were identified from the control cells, while 3745 proteins were identified from the gigantol-treated cells. The protein lists from the control and gigantol-treated cells were input to a Venn diagram and 2373 proteins (54.54%) were identified as being only from the control cells, 1767 proteins (47.18%) only from the gigantol-treated cells, and 1978 proteins from both groups (Figure 2A). The protein lists that were uniquely found in the control or gigantol-treated cells were subjected to further bioinformatic analysis (the lists of proteins are in Table S1).

The down- and upregulated protein lists were categorized into three ontologies, molecular function, biological process, and cellular component using Panther software (conducted on 8 October 2019). The most overrepresented molecular functions were binding (38.3% down- and 35.6% upregulated proteins) and catalytic activity (32.0% down- and 35.6% upregulated proteins) (Figure 2B). The most overrepresented biological process categories were cellular process (31.8% down- and 32.6% upregulated proteins), and metabolic process (21.0% down- and 19.8% upregulated proteins) (Figure 2C). The most overrepresented cellular component categories were cell (39.4% down- and 40.2% upregulated proteins) and organelle (33.6% down- and 32.2% upregulated proteins) (Figure 2D).

The two protein lists were input to Enrichr software to identify the enriched biological processes associated with the down- and upregulated proteins after treating with gigantol (conducted on 8 October 2019). Enrichment terms from the Gene Ontology (GO) biological process of downregulated proteins in gigantol-treated cells are involved in macromolecule biosynthesis, DNA-templated transcription, gene expression, protein phosphorylation, cytoskeleton organization, and telomere maintenance. In contrast, enrichment biological processes of upregulated proteins in gigantol-treated cells are involved in intracellular signal transduction, protein phosphorylation, gene expression, and protein biosynthesis processes (Table 1; The full lists of the enriched biological processes of down- and upregulated proteins altered by gigantol are in Tables S2 and S3).

### 2.3. Protein–Protein Interaction Networks of the Down- and Upregulated Proteins in Gigantol-Treated Cells

Kinases are vital enzymes that regulate intracellular signaling. Several oncogenes and tumor suppressor genes are kinase enzymes or proteins linked to protein kinase activity. Therefore, the proteins that linked to the GO term “protein phosphorylation (GO:0006468)” obtained from Enrichr were subjected to protein-protein interaction network analysis with the Search Tool for Retrieval of Interacting Genes/Proteins (STRING) database in order to determine the significant kinase pathways affected by gigantol.

**Figure 3:**
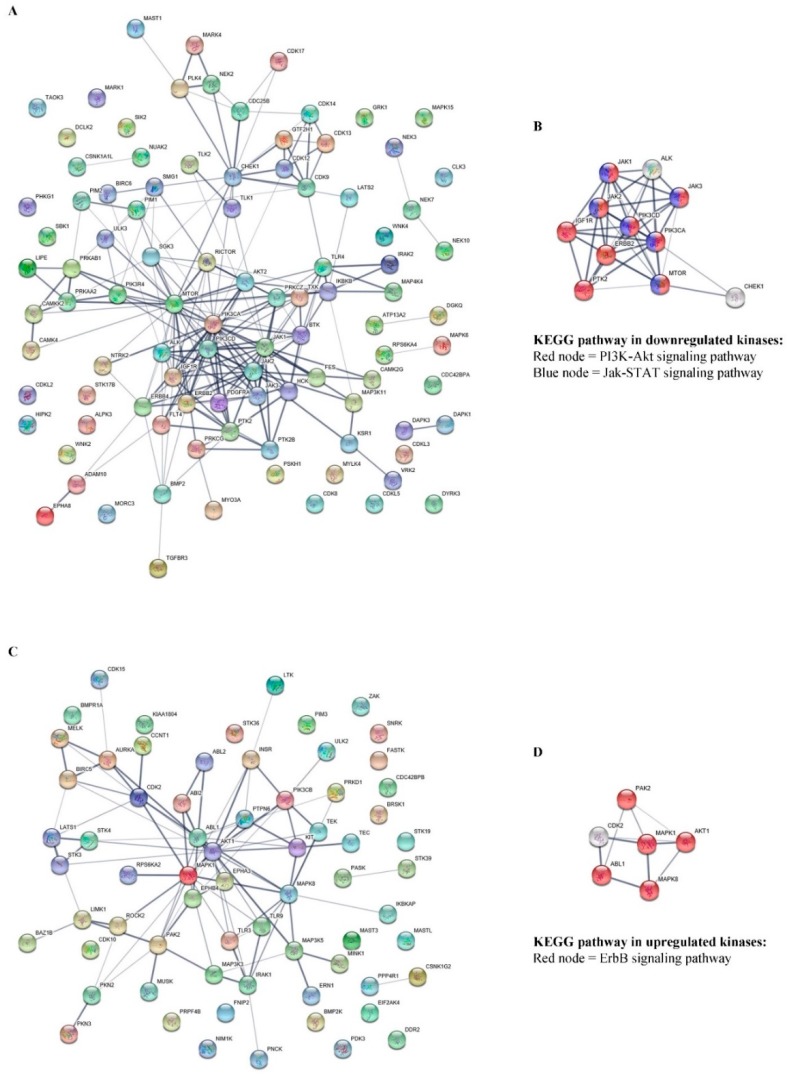
Networks presenting the functional protein-protein interactions of the (**A**) 97 down- and (**C**) 67 upregulated proteins related to the GO term “protein phosphorylation” (GO:0006468). The significant nodes of each network are identified and rebuilt as a network of CSC linked pathways. (**B**) According to the KEGG pathways database, significant nodes of the downregulated proteins were labeled with red for the PI3K-AKT signaling pathway (hsa04151) with FDR 4.08e−13 and blue for the JAK-STAT signaling pathway (hsa04630) with false discovery rate (FDR) 2.80e−09. (**D**) Significant nodes of upregulated proteins were labeled with red for the ErbB signaling pathway (hsa04012) with FDR 1.28 × 10^−9^

The 97 proteins that were downregulated and 67 proteins that were upregulated in gigantol-treated cells obtained from the GO term “protein phosphorylation (GO: 0006468)” were separately input to the STRING software (conducted on 8 October 2019). The resulting networks were presented (Figure 3A,C), and the significant nodes were determined from both the down- and upregulated protein lists. The top 10% of the downregulated proteins that had the most protein interactions were the following: MTOR, PIK3CA, JAK1, JAK2, PIK3CD, ERBB2, CHEK1, IGF1R, PTK2, ALK, and JAK3. Whereas, the top 10% of the upregulated proteins that had the most protein interactions were the following: AKT1, MAPK1, ABL1, MAPK8, CDK2, and PAK2.

The significant nodes of downregulated proteins were then analyzed for the pathways involved in CSCs using STRING, as shown in Figure 3A. According to the Kyoto Encyclopedia of Genes and Genomes (KEGG) pathway database (https://www.genome.jp/kegg/), the significant proteins that were downregulated by gigantol treatment were involved primarily in the PI3K/AKT and JAK/STAT signaling pathways (Figure 3B). These pathways were indicated as signaling pathways regulating the pluripotency of stem cells. Whereas, the significant nodes of the upregulated proteins were related to the ErbB signaling pathway, which supported the CSCs’ properties (Figure 3D). Interestingly, gigantol was previously shown to potentially suppress cancer cells growth, anoikis-resistance, and cancer stemness [25,26,27]. Regarding the data, the downregulated kinase proteins that were affected by gigantol treatment were linked to the many well-known pathways associated with tumorigenesis and CSC maintenance.

**Figure 4:**
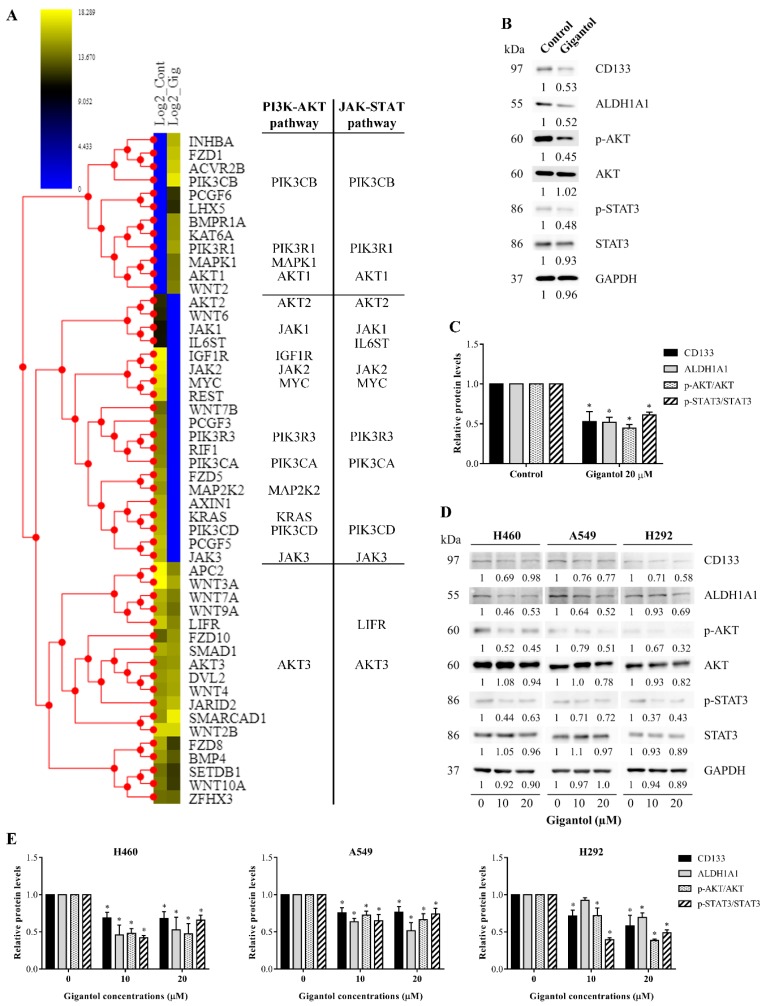
(**A**) Heatmap representing the levels of proteins associated with the signaling pathways regulating the pluripotency of stem cells in the control and gigantol-treated H460 cells (left and right columns of the heatmap, respectively). Proteins belonging to each pathway are listed to the right. (**B**) CSC markers and key kinases of AKT and STAT3 were determined by Western blotting and (**C**) the immunoblot signal intensities were quantified by densitometry. The uncropped protein bands are in Figure S2 (S2A: The protein bands from Figure 4B; S2B: The protein bands from Figure 4D). (**D**) The effects of gigantol on AKT, STATS3, and CSC markers were confirmed in two other NSCLC cell lines, A549 and H292, and (**E**) the relative protein levels were quantified. The mean data from each experiment was normalized to the GAPDH results. The experiment was performed biologically triplicated. Data represent the means ± SD (*n* = 3) * indicates *p* < 0.05 as compared with the control group (student’s *t*-test).

We further confirmed that the gigantol target pathways were crucial for CSC maintenance. The list of proteins involved in CSC regulation was extracted from the KEGG pathway database, using the term “hsa04550: Signaling pathways regulating pluripotency of stem cells—Homo sapiens (human)”, and mapped with our H460 proteomic profiles (conducted on 28 October 2019.). In total, 50 proteins were represented in the KEGG pathway, 12 proteins were significantly upregulated, 20 proteins were significantly downregulated, and 18 proteins were not significantly altered by gigantol (Figure 4A). Remarkably, the downregulated proteins affected by gigantol were mostly linked to the PI3K/AKT and JAK/STAT pathways (protein lists of the two pathways were obtained from KEGG pathway database “hsa04151: PI3K-AKT signaling pathway—Homo sapiens (human)” and “hsa04630: JAK-STAT signaling pathway—Homo sapiens (human)”; conducted on 28 October 2019.). Nevertheless, the levels of proteins belonging to the Wnt pathway, another pathway related to CSCs, were not significantly changed.

To confirm, the key proteins of PI3K/AKT and JAK/STAT pathways including AKT, phosphorylated Akt (S473), STAT3, phosphorylated STAT3 (S727), and CSC markers were determined by Western blot analysis using the same cell population of proteomics and xenograft experiments. The band density of active form of Akt (phosphorylated AKT) and active STAT3 (phosphorylated STAT3) was normalized with their own total forms in order to determine the levels of activation. The results showed that gigantol could inhibit the activation of AKT and STAT3. In addition, the CSC makers (CD133 and ALHD1A1) were found to be significantly reduced in response to gigantol treatment (Figure 4B,C). Moreover, the effect of gigantol on PI3K/AKT, JAK/STAT, and CSC markers was confirmed in other NSCLC cells (A549 and H292 cells) (Figure 4D,E). It was quite clear that the PI3K/AKT and JAK/STAT signaling pathways were the target pathways of gigantol on CSC maintenance in NSCLCs. It was possible that gigantol could show some effects on tumor formation and its integrity in vivo by means of CSC suppression.

### 2.4. Gigantol Negatively Regulates Tumor Cell Growth in Vivo

The concept of the in vivo xenograft was to compare the ability to form and maintain a tumor between the untreated control and gigantol-treated H460 cells. This experiment revealed the effects of gigantol on the cancer cells whether the CSC or other survival signals were suppressed by the treatment at the time of inoculation. The pretreatment procedure excluded the direct anticancer effect of the compound on the tumor cell after mice implantation.

**Figure 5:**
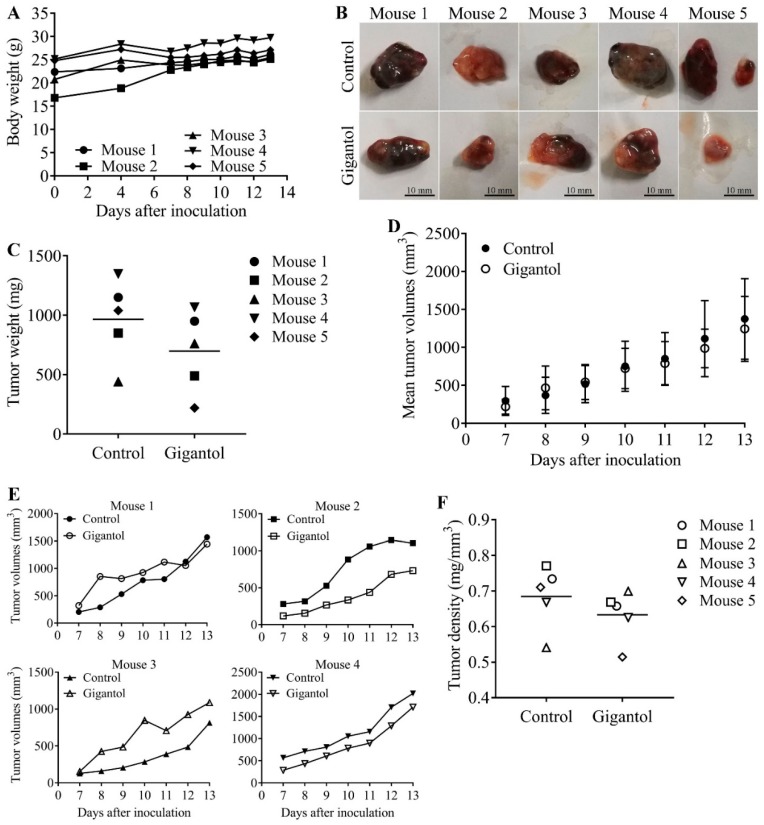
(**A**) Graph showing mice body weights starting at the day of cancer cell inoculation. There was no significant change of the body weights until the day of termination. (**B**) Untreated (upper row) and gigantol-treated (lower row) tumors were dissected and photographed at day 13 after inoculation. Scale bars represent 10 mm in length. (**C**) Graph showing grouped means of the control and gigantol tumor weights. The 5 different markers represent each pair of tumors. The gigantol-treated tumors had lower tumor weights as compared with their own control tumors, except for mouse 3. (**D**) Graph presenting the mean growth rate of the control and gigantol groups. (**E**) Four graphs demonstrating the individual tumor growth rate of each mouse (tumor growth of mouse 5 could not be accomplished because the gigantol-treated tumor was not palpable and measured until the day of termination). (**F**) Tumor density was calculated as weight by volume. The horizon lines represent means of each group.

After injection of lung cancer cells into two flanks of each mouse, most mice generated palpable tumors on day seven and most control tumors had reached their endpoint size on day 13. Figure 5A demonstrates that every mouse had a similar growth rate (indicated by body weight) in a normal range. The results showed that most gigantol-treated tumors were lighter than their paired control tumors (Figure 5B,C). However, the average weights of the tumors were only slightly different (control group mean = 966 ± 154.4 mg, gigantol group mean = 698 ± 154.5 mg, *n* = 5, *p* = 0.255, Student’s *t*-test). Tumor growth rates varied between the mice, but the mean tumor growth rates of the control and gigantol groups were not different (Figure 5D,E). The dissected tumor densities were compared, and the results showed that the gigantol groups had lower tumor densities than their paired untreated controls (Figure 5F).

### 2.5. Histological Observation Showed Lower Viable Tumor Areas in the Gigantol-Treated Tumors

**Figure 6:**
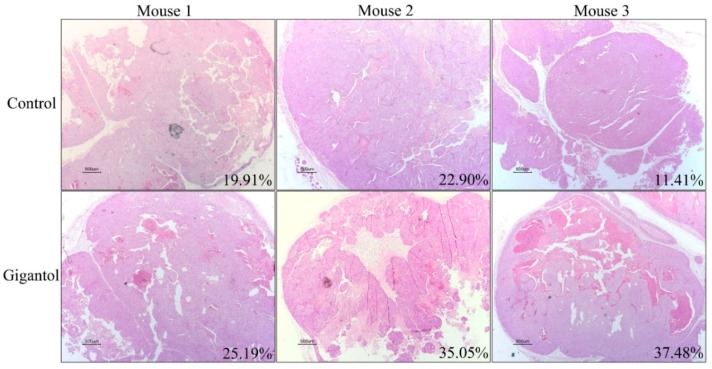
Hematoxylin and eosin staining showing intratumor morphology (20×). Percentages of necrotic areas as compared with their total areas are shown at the lower-right edge of each picture. Scale bars at the lower-left edge of each picture represent 500 µm lengths.

Having shown that gigantol pretreatment caused tumors with a lesser density as compared with the untreated control, we wish to emphasize this phenomenon as previous studies have indicated that changes in tumor density, as indicated by CT imaging showing a loss of tumor mass, can be a potential assessment for anti-cancer drug action [29,30]. Cross-section slices of the tumors were co-stained by hematoxylin and eosin (H&E), and, then, were photographed. The macro-morphology of the tumor structure was similar among all the tumors (small nodules packed within a tumor lobe, surrounded with fibrous tissues), whereas the percentage of intact and non-viable tumor cells of the two groups were dramatically different. Figure 6 demonstrates that while the control tumors exhibited a dense viable tumor mass, the gigantol tumors showed a substantial loss of tumor mass, as indicated by a hollowing with the magenta staining of cells or pale pink cells without nuclear staining.

### 2.6. Gigantol Suppresses Tumor Cells Proliferation but not Tumor Vasculature.

**Figure 7:**
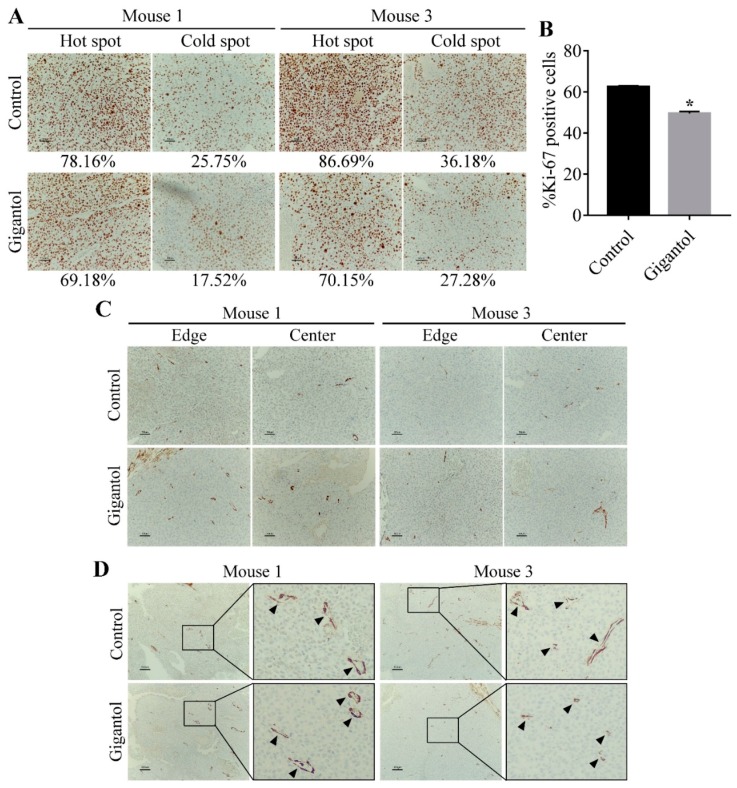
(**A**) Immunohistochemistry (IHC) staining demonstrating 200-fold magnified pictures of hot spots and cold spots from the control and gigantol-treated tumors. The percentages of Ki-67 positive cells as compared with total cells are displayed under their pictures. (**B**) Graph showing the means of %Ki-67 positive cells. The gigantol-treated tumors have lower Ki-67 positive cells than the control tumors. * indicates *p* < 0.05 as compared with the control group (Student’s *t*-test). (**C**) α-SMA IHC staining of cancer cells in both edge and center areas of tumors showing no difference of signal levels (200×). The numbers of mature tumor vessels per areas between the control and gigantol groups were not different. (**D**) Pictures showing vessel distribution among the tumor mass (100×). Arrow indicates a vessel (400×).

Two pairs of tumors were selected for Ki-67 and α-smooth muscle actin (α-SMA) immunohistochemistry (IHC) staining. The hot spots and cold spots of Ki-67 positive cells are shown in Figure 7A. The mean percentage of Ki-67 positive cells of the control group was 62.45 ± 0.3951 and that of the gigantol-treated group was 49.49 ± 0.7348 (*p*-value = 0.0041, Student’s *t*-test, *n* = 2, Figure 7B).

The α-SMA signals from cancer cells in all tumors were so low that they could not be scored (Figure 7C). This result indicated that both the control and gigantol groups had a low level of mesenchymal-like phenotypes. Figure 7D presents mature vessels covered by pericytes. The number of vessels per area detected by α-SMA staining was similar in all the tumors.

## 3. Discussion

According to the increasing trend of cancer incidence and mortality, the development of novel anti-cancer therapies is highly needed. Among malignant tumors, lung cancer has been shown to be the main cause of cancer-related mortality and treatment failure [31], leading to the requirement for effective therapeutic options. Previous studies have reported the potential anti-cancer activities of gigantol, one of the most widely studied bibenzyls. Gigantol has exhibited cytotoxicity against various types of cancer cells, such as breast, liver, and lung cancer cells [24,32,33]. Moreover, gigantol could attenuate certain aggressive phenotypes that bring about tumor progression and metastasis, including proliferation, migration, invasion, anoikis-resistance, and anchorage-independent growth [25,26,27,28].

Proteomics analysis (Figure S1).demonstrated that PI3K/AKT/mTOR and JAK/STAT were among the most affected proteins in response to gigantol treatment. The key kinases belonging to the PI3K/AKT/mTOR axis, including phosphoinositide 3-kinases (PI3Ks, α and δ isoforms) and mammalian target of rapamycin (mTOR), were significantly decreased in the gigantol-treated cells (Figure 3A,B). Both isoforms of PI3K can activate phosphatidylinositol (3,4,5)-trisphosphate (PIP3), an upstream activator of AKT [34]. In addition, PI3K can trigger an AKT-independent mechanism, which transduces signals through serine/threonine-protein kinase Sgk3 (SGK3) and mTOR complex 2 (mTORC2) [35]. Consistently, our proteomic results showed the suppression of key proteins of the PI3K-mediated AKT-independent pathway, such as PIK3CA, PIK3CD, SGK3, MTOR, and RICTOR, which were simultaneously downregulated (Table S1).

Janus kinase 1 and 2 (JAK1 and JAK2) are transducers of the heteromeric receptors of interleukin 6 and 10 (IL-6 and IL-10), which activate signal transducer and activator of transcription 3 (STAT3). STAT3 was shown to mediate cancer cell survival, proliferation, angiogenesis, and metastasis, as well as maintaining the CSC phenotypes [7,36]. Although the STAT3 protein could not be detected in our proteomic profiles due to its low abundance, its downstream target genes, including cyclin D1 and c-Myc, were downregulated [12] (Table S1). JAK3 is an upstream regulator of STAT5 and STAT6. An accumulating data exercise revealed that inhibition of the JAK3 signaling could reduce cancer progression [37]. It is possible that the suppression of JAK/STAT signaling by gigantol should attenuate CSC in lung cancer. In addition, the mitogen-activated protein kinase 8 (MAPK8) or c-Jun N-terminal kinase (JNK) protein level was found to be induced by gigantol treatment (Figure 3D). JNK plays a role in controlling cancer cell death. The activation of JNK is necessary for intrinsic and extrinsic apoptosis, and autophagic cell death [38]. JNK signaling has been reported as a vital molecular mechanism of many anti-cancer-agents-induced cancer cell death and inactivation of such a protein led to cancer cell resistance to death stimuli [39,40]. An early upregulation of JNK by gigantol before the cancer cells encountered the stressful conditions in the tumor possibly led to stress induced JNK hyperactivation, which subsequently promoted the expression of proapoptotic proteins [38].

Recent evidence has suggested that CSCs functions as a seed of tumors. Not only do the CSCs use their ability of self-renewal and differentiation for tumor establishing, but also implicate cancer progression, metastasis, and disease relapse [1]. Regarding this matter, our previous work unraveled new information that gigantol could suppress CSC activity and discontinue their role in maintaining tumor [2,41]. This finding is quite in agreement with the previous study indicating that CSCs play a key role in tumor maintenance. This study revealed the effect of gigantol of PI3K/AKT and JAK/STAT3 suppression on the tumor initiation, growth, and maintenance based on the concept that the cells at the first step of tumor initiation had lesser CSC property than the control untreated cells.

In this study, the lung cancer cells were treated with a noncytotoxic concentration of gigantol prior to inoculation into mice subcutaneous skins. The same populations of gigantol-treated cells were subjected to proteomics. This experimental design displays the clear mechanism of gigantol treatment in attenuation of the CSC-supportive PI3K/AKT/mTOR and JAK/STAT3 signals at the time of tumor initiation. Although this experiment used only a single treatment with low dose, gigantol could inhibit the tumor growth rate (Figure 5E). Tumors from the gigantol-pretreated cells had lower weights and densities (Figure 5C,F). Furthermore, the histological tumor integrity was determined. Previous studies have either demonstrated or proposed that the tumor density can be a promising assessment of anti-cancer drug evaluation as they have given more accuracy on the assessment of an anti-cancer drug response and have contributed to better treatment outcomes [29,30,42]. We observed the cross-sectional histology of the tumors to assess the integrity of intact cell viable areas as compared with the cell death areas as recommended in the guideline [43]. Interestingly, our results indicated that most of the gigantol-treated tumors had a dramatic loss of tumor mass as compared with those of the untreated controls (Figure 6). Consistently, the intratumor structure and tumor phenotype of Ki-67 labeling showed that the gigantol-treated tumors had lower proliferative cancer cells (Figure 7A,B). However, we found that the EMT properties of cancer cells observed by α-SMA staining was not altered by treatment with gigantol (Figure 7C). Also, the angiogenic capability of both groups of tumors was not different (Figure 7D). The phenotypic observation revealed that the pretreatment with gigantol did not have an effect on tumor neoangiogenesis. Further investigation on gigantol-mediated stromal cell-induced angiogenesis is thus suggested.

This study was designed in a manner of a pharmacological study that minimized the confounding factors in the system and focused on the effect of gigantol on the cancer cells. We could assume from the results that gigantol treatment altered the tumor-promoting activities of the cells prior to the process of tumor inoculation and such alteration attenuated the ability of the cancer cell to grow and maintain a tumor, resulting in a reduced tumor mass with viable cancer cell loss. Although our results helped us scope the direct action of the compound on NSCLCs, further investigation is necessary, including the injection of the substance into a tumor or animal after tumor formation to gain more insights.

## 4. Materials and Methods

### 4.1. Cell Line Cultures

Human NSCLC H460 and normal bronchus epithelial cell BEAS-2B lines were purchased from the American Type Culture Collection (Manassas, VA, USA) and were cultured in Roswell Park Memorial Institute (RPMI) 1640 medium and Dulbecco’s modified Eagle medium (DMEM), respectively, supplemented with 10% fetal bovine serum, 2 mM L-glutamine, and 100 units/mL each of penicillin and streptomycin in a humidified atmosphere with 5% CO_2_ at 37 °C.

### 4.2. Animals

Six-week old male BALB/cAJcl nude mice were purchased from Nomura Siam International (Samut Prakan, Thailand). Five mice were maintained in one cage under strictly hygiene housing with controlled temperature (23 ± 2 °C) and light/dark cycle (12 h light/12 h dark) at the Animal House of Faculty of Medicine, Chulalongkorn University. The study was approved by the Institutional Animal Care and Use Committee of the Faculty of Medicine, Chulalongkorn University, Bangkok, Thailand (ethical reference number CULAC 001/2561). Animal welfare and experimental procedures were strictly carried out in accordance with The Eighth Edition of the Guide for the Care and Use of Laboratory Animals (NRC 2011) [44]. All efforts were made to minimize animals’ suffering and to reduce the number of animals used.

### 4.3. Chemicals and Reagents

Gigantol was extracted from stems of Dendrobium draconis Rchb.f., as previously described [45] and dissolved in dimethylsulfoxide (DMSO) at the indicated working concentrations. 3-(4,5-Dimethylthiazol-2-yl) 2,5-diphenyltetrazolium bromide (MTT), Hoechst 33342, propidium iodide (PI), bovine serum albumin (BSA), dimethyl sulfoxide (DMSO), cocktail protease inhibitor, hematoxylin, and eosin were purchased from Sigma chemical, Inc. (Chemical Express, Bangkok, Thailand). RPMI-1640 medium, DMEM, phosphate buffer saline (PBS), glutamine, penicillin, and streptomycin were purchased from Gibco company (Gibthai, Bangkok, Thailand). Primary antibodies against CD133, ALDH1A1, total AKT, phosphorylated AKT (Ser473), total STAT3, phosphorylated STAT3 (Ser727), and GAPDH, horseradish peroxidase labeled secondary antibodies, and RIPA lysis buffer were purchase from Cell Signaling Technology (Theera Trading, Bangkok, Thailand). Pentobarbital sodium injection was purchased from Ceva Sante Animal (VET AGRITECH, Nonthaburi, Thailand). 3,3′-Diaminobenzidine tetrahydrochloride hydrate was purchased from TCI Co., LTD (Chemical Express, Bangkok, Thailand). Primary antibodies of Ki-67 and α-SMA and matched secondary antibodies were purchased from DAKO (Medicare Supply, Bangkok, Thailand).

### 4.4. Cell Viability Assay

In order to elucidate the possible tumor suppression activity of gigantol, first, we selected the concentrations of the compound that caused no toxicity to the cancer cells. Cell viability was determined by plating cells at a density of 10,000 cells per well in 96-well plates. The cells were allowed to adhere overnight, medium was removed, and medium with various concentrations of gigantol (0 to 200 µM) or 0.1% DMSO was added. After 24 to 48 h of treatment, the number of viable cells were measured with the use of MTT assay. Medium was aspirated and 0.4 mg/mL of MTT in PBS was added to each well. The plate was then incubated at 37 °C, 5% CO2 for 3 h. Afterwards, the resulting formazan crystal was dissolved in 100 µL of DMSO and subjected to a 570 nm absorbance reading via a microplate reader (ClarioStar, BMG Labtech, Germany). The assay was performed biological triplicate.

### 4.5. Cell Death Determination Assay

Nuclear co-staining with Hoechst 33342 and propidium iodide (PI) was used to determine apoptotic and necrotic cell death. Cells were treated with gigantol as described in cell viability assay. Then, cells were incubated with 10 µM of Hoechst 33342 and 5 µM PI for 30 min at 37 °C. Cells were visualized and imaged under a fluorescence microscope (Nikon eclipse Ts2 with Nikon DS Fi3 camera). Apoptotic cell could be detected by Hoechst 33342 nuclear staining, showing condensed nucleus and fragmented nuclei of apoptotic bodies. Necrotic cell could be detected by PI staining.

### 4.6. Sample Preparation

H460 cells were treated with 20 µM gigantol or 0.01% DMSO (vehicle) for 24 h. The cells were lysed with 0.5% SDS. Total protein amount collected from each sample was measured with Lowry assay with bovine serum albumin as a standard [46]. Equal protein amount from 3 independent biological samples were pooled. Fifty micrograms of protein from control or gigantol treated cells were subjected to in-solution digestion. Samples were completely dissolved in 10 mM ammonium bicarbonate (AMBIC), reduced disulfide bonds using 5 mM dithiothreitol (DTT) in 10 mM AMBIC at 60 °C for 1 h and alkylation of sulfhydryl groups by using 15 mM Iodoacetamide (IAA) in 10 mM AMBIC, at room temperature for 45 min in the dark. For digestion, samples were mixed with 50 ng/µL of sequencing grade trypsin (1:20 ratio) (Promega, Walldorf, Germany) and incubated at 37 °C overnight. Prior to LC-MS/MS analysis, the digested samples must be dried and protonated with 0.1% formic acid before injection into LC-MS/MS.

### 4.7. Liquid Chromatography-Tandem Mass Spectrometry (LC-MS/MS)

The LC-MS/MS was used to determine the quantification of the peptides from the digested samples. The tryptic peptide samples were prepared for injection into an Ultimate3000 Nano/Capillary LC System (Thermo Scientific, Gloucester, UK) coupled to a Hybrid quadrupole Q-Tof impact II™ (Bruker Daltonics, Coventry, UK) equipped with a Nano-captive spray ion source. Briefly, peptides were enriched on a µ-Precolumn 300 µm i.d. × 5 mm C18 Pepmap 100, 5 µm, 100 A (Thermo Scientific, UK), separated on a 75 μm I.D. × 15 cm and packed with Acclaim PepMap RSLC C18, 2 μm, 100Å, nanoViper (Thermo Scientific, UK). Solvent A and B containing 0.1% formic acid in water and 0.1% formic acid in 80% acetonitrile, respectively, were supplied on the analytical column. A gradient of 5% to 55% solvent B was used to elute the peptides at a constant flow rate of 0.30 μL/min for 30 min. Electrospray ionization was carried out at 1.6 kV using the CaptiveSpray. Mass spectra (MS) and MS/MS spectra were obtained in the positive-ion mode over the range (m/z) 150–2200 (Compass 1.9 software, Bruker Daltonics).

### 4.8. Bioinformatics and Data Analysis

MaxQuant 1.6.6.0 was used to quantify the proteins in individual samples using the Andromeda search engine to correlate MS/MS spectra to the Uniprot Homo sapiens database [47]. The following parameters were used for data processing: maximum of two miss cleavages, mass tolerance of 20 ppm for main search, trypsin as digesting enzyme, carbamidomethylation of cysteine as fixed modification, and the oxidation of methionine and acetylation of the protein N-terminus as variable modifications. Only peptides with a minimum of 7 amino acids, as well as at least one unique peptide, were required for protein identification. Only proteins with at least two peptides, and at least one unique peptide, were considered as being identified and used for further data analysis.

The gene list enrichment analysis was conducted using Enrichr software (https://amp.pharm.mssm.edu/Enrichr/) [48]. Protein organization and biological action was investigated conforming to protein analysis through evolutionary relationships (Panther software; http://pantherdb.org/) protein classification [49]. A Venn diagram (analyzed by jVenn software; http://jvenn.toulouse.inra.fr/app/index.html) was used to show the differences between protein lists originating from different differential analyses [50]. The Search Tool for Retrieval of Interacting Genes/Proteins (STRING) software version 11 (https://string-db.org/cgi/input.pl) was used to analyze the common and the forecasted functional interaction networks between identified proteins [51]. Cytoscape 3.7.2 (https://cytoscape.org/) was utilized to analyze the significant nodes from protein–protein interaction networks [52]. The significant nodes analysis was modified from Rezaei-Tavirani (2017) [53]. The degree values, which were determined by an amount of interacted proteins with the node, were analyzed and the top 10% of the nodes based on degree value were selected as significant nodes. The heatmap visualization and statistical analyses were conducted using the MultiExperiment Viewer (MeV) in the TM4 suite software (http://mev.tm4.org/#/welcome) [54].

### 4.9. Western Blot Analysis

Cells were lysed with RIPA lysis buffer containing 20 mM Tris-HCl (pH 7.5), 150 mM NaCl, 1 mM Na_2_EDTA, 1 mM EGTA, 1% NP-40, 1% sodium deoxycholate, 2.5 mM sodium pyrophosphate, 1 mM beta-glycerophosphate, 1 mM Na_3_VO_4_, 1 µg/mL leupeptin, and cocktail protease inhibitor mixture for 30 min on ice. The protein contents of the cell lysates were evaluated by Lowry assay. Samples with equal amounts of protein (60 µg) were run in the SDS-PAGE before they were transferred onto 0.45 mm nitrocellulose membranes (Bio-Rad, Hercules, California, United States). Transferred membranes were blocked for 1 h in 5% non-fat dry milk in Tris-buffered saline with Tween 20 (25mM Tris-HCl, pH 7.5, 125 mM NaCl, and 0.05% Tween 20) and incubated overnight with specific primary antibodies against CD133, ALDH1A1, total AKT, phosphorylated AKT (Ser473), total STAT3, phosphorylated STAT3 (Ser727), and GAPDH. Membranes were washed three times with Tris-buffered saline with Tween 20 and incubated with appropriate horseradish peroxidase labeled secondary antibodies for 2 h at room temperature. The immune complexes were detected by Clarity and Clarity Max ECL Western Blotting Substrates (Bio-Rad) and imaged with ImageQuant LAS 4000 biomolecular imager (GE Healthcare, Chicago, Illinois, United States).

### 4.10. Subcutaneous Tumor Xenograft Procedure

**Figure 8:**
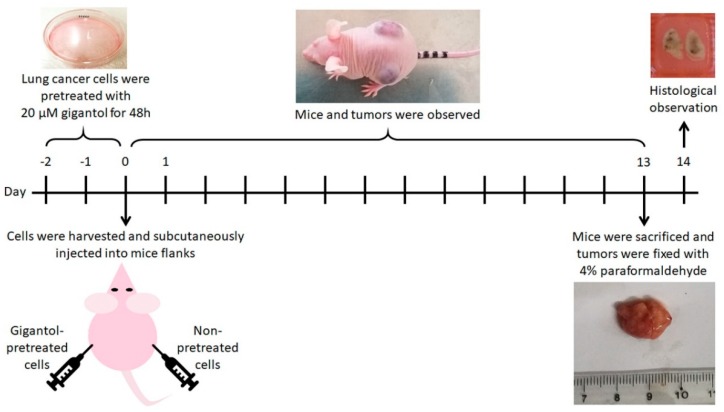
Scheme showing the in vivo experimental procedures.

The scheme of experimental design is shown in Figure 8. The human NSCLCs were prepared prior to the tumor establishment. The H460 cells were cultured in medium with 20 µM of gigantol or vehicle for 48 h (5 individual sets of cancer cell cultures). Then, the 70% confluent monolayer lung cancer cells were trypsinized, suspended in Hank’s saline buffer solution and counted by TC20 automated cell counter (Bio-Rad). Each cell suspension was adjusted to a concentration of viable 5 × 106 cells per 100 µL. The cancer cell suspensions were kept on ice and rapidly transferred to an in vivo subcutaneous xenograft operation.

To minimize variation between animal bodies, one mouse was assigned to bear both control and its paired gigantol-treated tumor. One flank of a mouse was inoculated subcutaneously with viable 5 × 106 cells of untreated cells and another flank with gigantol pretreated cells. Mice were weighed, and the tumors were observed every 2 days. When a tumor was palpable, the mouse would be observed daily. Vernier Caliper was used to measure the most length and its own orthogonal most width of each tumor. Tumor volumes were calculated by the formula (length × width × width)/2. Tumor growth rates were verified by means of plotting calculated tumor volumes by days. Mice were not exposed to gigantol throughout the experiment. Once control or treatment tumor reached its endpoint size (20 mm in diameter), the tumor-bearing mouse was euthanized by intraperitoneal injection of pentobarbital sodium solution (>150 mg/kg) [55] and then the tumors were dissected, washed with ice-cold PBS, weighed, and photographed with a ruler. The tumors were weighed and immediately fixed with 4% paraformaldehyde for 24 h. Tumors were embedded in paraffin blocks, sliced and stained with hematoxylin and eosin (H&E) for further histologic observation. Necrotic and total areas of tumor slices were determined using ImageJ software [56].

### 4.11. Immunohistochemistry Staining of Ki-67 and α-SMA

Two pairs of tumor slides were selected for staining with Ki-67 (dilution 1:300) and α-smooth muscle actin (α-SMA, dilution 1:100) antibodies and, then, were visualized by incubation with 3,3′-diaminobenzidine. Slides were observed under a brightfield microscope (Nikon eclipse E600 with Nikon DXM1200F camera).

The level of Ki-67 assessment was modified from Jang (2017) [57]. Areas with the highest (hot spot) and the lowest (cold spot) numbers of positive cells (indicated by dark brown staining in nucleus) were selected and the percentages of the positive cells as compared with total cells were calculated. Averages of %Ki-67 positive cells were calculated from the summed total and Ki-67 positive cells from all hot spots and cold spots of the two mice.

α-SMA, a marker of mesenchymal phenotype, was used to detect cancer cells with EMT-like phenotype, endothelial cells, and vascular pericytes [58]. For angiogenesis determination, edge and center areas of each tumor were selected and the number of mature blood vessels (indicated by circular lining of cells labeled with high signal of α-SMA) were counted.

### 4.12. Statistical Analysis

One-way analysis of variance (one-way ANOVA) and student’s *t*-test were performed to conduct statistical analysis (GraphPad Prism 7.0). Data were expressed as mean ± standard deviation (SD) and values of *p* < 0.05 were indicative of significant differences.

## 5. Conclusions

Data from this study demonstrated that pretreatment with gigantol can suppress tumor growth, reduce tumor density, and attenuate the tumor maintenance of NSCLCs. This information can benefit and encourage further investigation of this useful compound to be used for anti-cancer approaches.

## Figures and Tables

**Table 1:** First 10 ranking enrichment terms from the GO biological process of down- and upregulated proteins in gigantol-treated cells.

Term	Overlap	*p*-Value
enriched biological processes associated with the down-regulated proteins		
regulation of cellular macromolecule biosynthetic process (GO:2000112)	129/632	2.65 × 10^–10^
regulation of nucleic acid-templated transcription (GO:1903506)	121/608	4.65 × 10^–9^
protein phosphorylation (GO:0006468)	97/471	2.82 × 10^–8^
regulation of transcription, DNA-templated (GO:0006355)	254/1599	3.09 × 10^–7^
regulation of gene expression (GO:0010468)	174/1038	9.47 × 10^–7^
phosphorylation (GO:0016310)	76/387	5.61 × 10^–6^
protein autophosphorylation (GO:0046777)	41/176	1.44 × 10^–5^
cytoskeleton organization (GO:0007010)	29/127	3.49E × 10^–4^
membrane depolarization during action potential (GO:0086010)	13/39	3.58 × 10^–4^
regulation of telomere maintenance (GO:0032204)	10/26	4.67 × 10^–4^
enriched biological processes associated with the up-regulated proteins		
regulation of intracellular signal transduction (GO:1902531)	62/423	4.82 × 10^–5^
protein phosphorylation (GO:0006468)	67/471	6.24× 10^–5^
ribosomal large subunit biogenesis (GO:0042273)	16/64	1.02 × 10^–4^
cyclic purine nucleotide metabolic process (GO:0052652)	10/31	2.17 × 10^–4^
regulation of gene expression (GO:0010468)	124/1038	2.75 × 10^–4^
nucleotide biosynthetic process (GO:0009165)	9/27	3.40 × 10^–4^
DNA replication checkpoint (GO:0000076)	7/17	3.59 × 10^–4^
RNA splicing, via transesterification reactions with bulged adenosine as nucleophile (GO:0000377)	37/237	4.55 × 10^–4^
regulation of mRNA processing (GO:0050684)	9/29	6.19 × 10^–4^
mRNA processing (GO:0006397)	42/284	6.22 × 10^–4^

## Supplementary Materials

The following are available online at https://www.mdpi.com/2072-6694/11/12/2032/s1, Figure S1: The workflow for proteomics analysis, Figure S2: The uncropped images of Western blot bands, Table S1: The lists of proteins which were downregulated, upregulated, and not significantly altered by the effects of gigantol, Table S2: GO biological process enrichment analysis results of the significantly downregulated proteins from Enrichr software, Table S3: GO biological process enrichment analysis results of the significantly upregulated proteins from Enrichr software.
