# Supplementary material for: Gigantol Targets Cancer Stem Cells and Destabilizes Tumors via the Suppression of the PI3K/AKT and JAK/STAT Pathways in Ectopic Lung Cancer Xenografts
Source: Cancers (Basel). 2019 Dec 17;11(12):2032. doi: 10.3390/cancers11122032 (PMC6966687; doi:10.3390/cancers11122032)
Supplement: Supplementary file 1 [file cancers-11-02032-s001.zip › cancers-647632-Supple-figures-final.docx]

Supplementary Materials: Gigantol Targets Cancer Stem Cells and Destabilizes Tumors via the Suppression of the PI3K/AKT and JAK/STAT Pathways in Ectopic Lung Cancer Xenografts

Nattanan Losuwannarak, Arnatchai Maiuthed, Nakarin Kitkumthorn, Asada Leelahavanichkul, Sittiruk Roytrakul and Pithi Chanvorachote


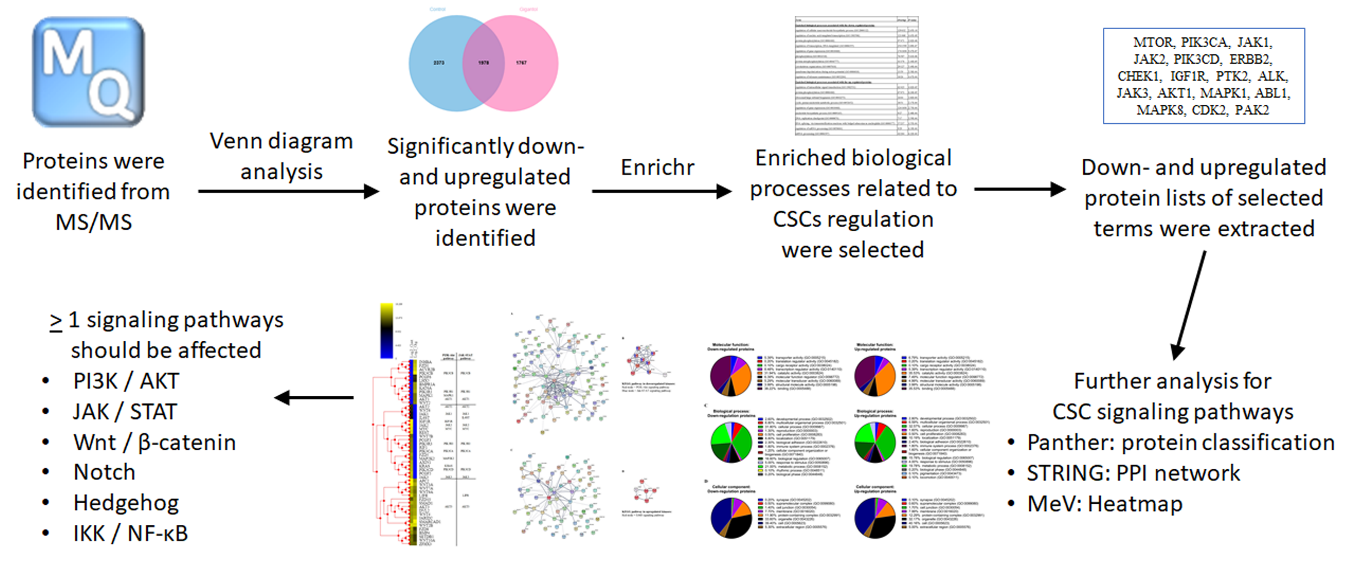


**Figure S1.** The workflow for proteomics analysis.


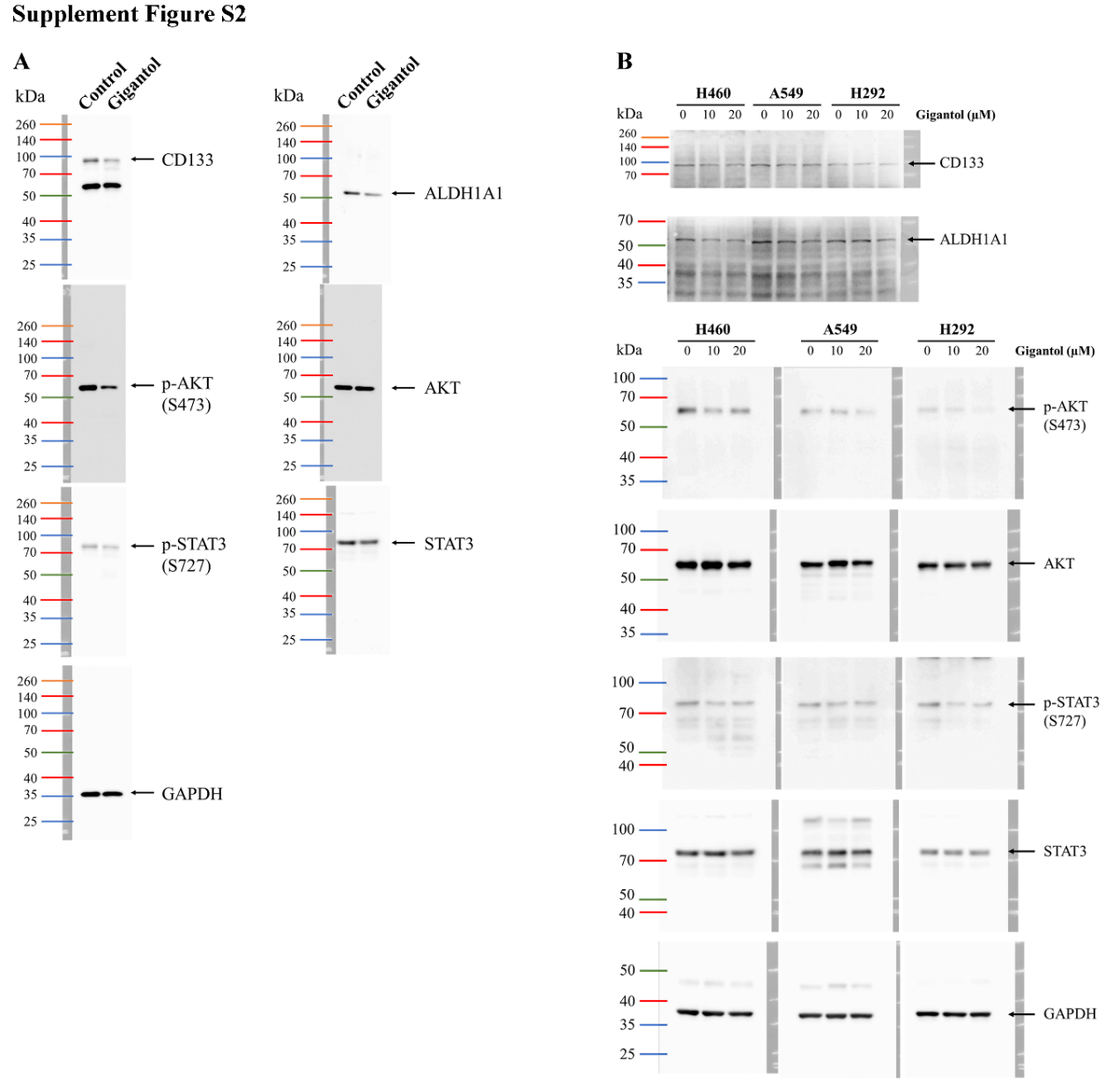


**Figure S2.** The uncropped images of Western blot bands.

| 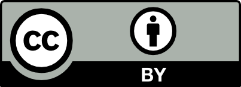 | © 2019 by the authors. Licensee MDPI, Basel, Switzerland. This article is an open access article distributed under the terms and conditions of the Creative Commons Attribution (CC BY) license (http://creativecommons.org/licenses/by/4.0/). |
| --- | --- |
